# Chemical syntheses and salient features of azulene-containing homo- and copolymers

**DOI:** 10.3762/bjoc.17.139

**Published:** 2021-08-24

**Authors:** Vijayendra S Shetti

**Affiliations:** 1 Department of Chemistry, National Institute of Technology Karnataka, Surathkal 575025, India

**Keywords:** azulene, chemical synthesis, copolymer, non-alternant hydrocarbon, organic electronics, polyazulene

## Abstract

Azulene is a non-alternant, aromatic hydrocarbon with many exciting characteristics such as having a dipole moment, bright color, stimuli responsiveness, anti-Kasha photophysics, and a small HOMO–LUMO gap when compared to its isomer, naphthalene. These properties make azulene-containing polymers an intriguing entity in the field of functional polymers, especially for organic electronic applications like organic field-effect transistors (OFET) and photovoltaic (PV) cells. Since azulene has a fused five and seven-membered ring structure, it can be incorporated onto the polymer backbone through either of these rings or by involving both the rings. These azulene-connection patterns can influence the properties of the resulting polymers and the chemical synthesis in comparison to the electrochemical synthesis can be advantageous in obtaining desired patterns of substitution. Hence, this review article presents a comprehensive overview of the developments that have taken place in the last three decades in the field of chemical syntheses of azulene-containing homo- and copolymers, including brief descriptions of their key properties.

## Introduction

**Figure 1:**
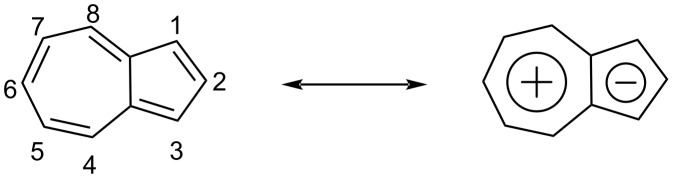
Chemical structure, numbering scheme, and resonance form of azulene.

Azulene (C_10_H_8_) is a non-alternant, non-benzenoid, 10 π electron aromatic hydrocarbon containing a fused seven- and five-membered ring [1–5]. The electron drift from the seven-membered ring to the five-membered ring is responsible for its polarized structure which features both a 6 π electron tropylium cation and a 6 π electron cyclopentadienyl anion in the same molecule (Figure 1).

The striking feature of azulene is its permanent dipole moment (1.08 D) and blue color unlike its colorless isomer naphthalene [4]. Azulene possesses an unequal distribution of electron density between its highest occupied molecular orbital (HOMO) and lowest unoccupied molecular orbital (LUMO) resulting in a relatively small electron repulsion energy in the first singlet excited state and thus, a small HOMO–LUMO (S_0_–S_1_) gap compared to naphthalene. The large energy gap between its S_2_ and S_1_ states (up to 15000 cm^−1^) makes internal conversion less probable, making azulene emit from the S_2_ state, violating Kasha’s rule [6]. These intriguing features have encouraged researchers to use azulene derivatives as functional organic molecules in the field of optoelectronics [7–14]. Employing such stimuli-responsive, non-alternant hydrocarbon with odd-membered rings in the chemical synthesis of functional polymers is also an interesting proposition and such polymers can find promising applications in the organic electronics field such as organic field-effect transistors (OFET) and photovoltaic (PV) cells [15–16]. The synthesis of azulene-containing polymers can be envisaged through chemical and electrochemical means. Through electrochemical methods, only the five-membered rings can be incorporated onto the polymer backbone, and often the polymers produced are insoluble [17]. On the other hand, chemical synthesis provides an avenue to synthesize soluble polymers where azulene can be incorporated into the backbone through either of the rings or by involving both the rings. These substitution patterns can influence the property of the resulting polymer. However, to achieve the synthesis of such homo- and copolymers, suitably tailor-made azulene monomers with specific substitution patterns are needed, and often the synthesis of such building blocks is challenging. This could be the reason why the chemical synthesis of azulene-containing polymers is only sporadically investigated. Hence, this article is intended to provide readers an overview of the developments in the area of chemical synthesis of azulene-containing homo- and copolymers that have been achieved during the last three decades.

## Review

### Azulene-containing homopolymers

#### The polyazulenes

**Scheme 1:**
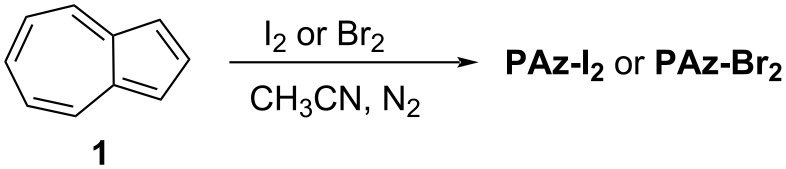
Synthesis of polyazulene-iodine (**PAz-I_2_)** and polyazulene**-**bromine (**PAz-Br_2_)** complexes.

The earliest chemical synthesis of polyazulene was reported by Neoh, Kang, and Tan in 1988 [18]. Their strategy was based on the oxidative polymerization of azulene (**1**) by iodine or bromine to obtain polyazulene–iodine/bromine complexes (**PAz-I_2_/PAz-Br_2_**) (Scheme 1).

The **PAZ-I_2_** complex was found to be insoluble whereas **PAZ-Br_2_** was sparingly soluble in most of the organic solvents. Although no structure was proposed for these polymer complexes, based on the elemental analysis, the authors provided the formula representation C_10_H_6_(I_2_)_0.4_ for **PAZ-I_2_** and C_10_H_4.9_(Br_2_)_0.72_ for **PAZ-Br_2_**. The gel permeation chromatography (GPC) analysis of the tetrahydrofuran (THF)-soluble fraction of **PAZ-Br_2_** indicated the presence of oligomeric species (average degree of polymerization of ≈7) leaving speculation about a higher degree of polymerization for the THF-insoluble component of **PAZ-Br_2_**. The UV–vis spectrum of the chloroform-soluble fraction of **PAZ-Br_2_** displayed absorption bands in the 190–330 nm region like the azulene monomer, and a long tail with a broad peak around 690 nm supporting the oligomeric nature of the soluble fraction. The thermogravimetric analysis (TG) showed that **PAZ-I_2_** was thermally more stable than **PAZ-Br_2_**. The electrical conductivity of **PAZ-Br_2_** (5 × 10^−3^ S/cm) was far too superior compared to **PAZ-I_2_** (10^−6^ S/cm).

**Scheme 2:**
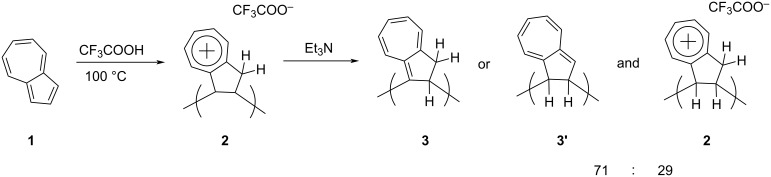
Synthesis of ‘true polyazulene’ **3** or **3’** by cationic polymerization.

In 1997, Kihara, Fukutomi, and Nakayama [19] reported the synthesis of what they described as ‘true polyazulene’ through a cationic polymerization reaction. Their protocol involved heating the trifluoroacetic acid (TFA) solution of azulene (**1**) followed by treatment with triethylamine to obtain a brown polymeric product called polyazulene (Scheme 2).

The elemental analysis of this polymer revealed that it existed as a 71:29 mixture of polymer **3** or **3’** bearing a heptafulvene structure (true polyazulene) and cycloheptatrienyl trifluoroacetate **2**. The temperature and reaction time determined the overall yield and the *M*_n_ of the polyazulene formed. The maximum yield of 89% and the highest *M*_n_ = 3600 Da was obtained when azulene was heated at 100 °C for 24 hours in TFA. However, changing the reaction medium from TFA to acetic acid or methane/trifluoromethane sulfonic acid did not facilitate the polyazulene formation. The polyazulene **3** or **3’** was soluble in various organic solvents such as toluene, dichloromethane, tetrahydrofuran (THF), and *N*,*N*’-dimethylformamide (DMF). The ‘true polyazulene’ exhibited the conductivity of 5.38 × 10^−8^ S/cm, which was increased to 8.16 × 10^−3^ S/cm upon exposure to iodine atmosphere, presumably due to the formation of dehydropolyazulene via oxidative aromatization.

**Scheme 3:**
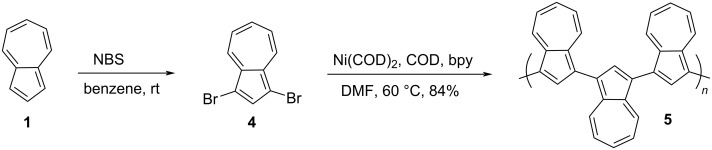
Synthesis of 1,3-polyazulene **5** by Yamamoto protocol.

In 2003, Lai and co-workers [20] synthesized 1,3-polyazulene **5** from azulene in two steps as shown in Scheme 3. First, azulene (**1**) was dibrominated at the 1,3-positions by treatment with *N*-bromosuccinimide (NBS) and the consequent dehalogenative polycondensation reaction of 1,3-dibromoazulene (**4**) by an organonickel catalyst (Yamamoto protocol) yielded 1,3-polyazulene **5**.

The polymer was partially soluble in many organic solvents such as chloroform, THF, xylenes, DMF, *N*-methylpyrrolidone (NMP), and also in Brønsted acids like TFA and conc. H_2_SO_4_. The molecular weight (*M*_n_) and polydispersity (PD) of 1,3-polyazulene **5** were 16,400 and 1.15, respectively, as determined by GPC in THF. The presence of resonance signals in the region δ 7.1–8.6 ppm of the ^1^H NMR spectrum and the close IR spectral resemblance to azulene confirmed the integrity of the azulene units in 1,3-polyazulene **5**. The robustness of the 1,3-polyazulene backbone was evidenced by TG analysis where it retained over 60% of its mass even after heating to 1000 °C. The absorption spectrum recorded in chloroform revealed a considerable red shift in the absorption band of 1,3-polyazulene **5** (417 nm) compared to azulene (**1**, 341 nm) supporting the presence of extended conjugation prevailing in the polymer. The absorption and EPR spectral patterns of 1,3-polyazulene **5** recorded in TFA and H_2_SO_4_ were contrasting to each other inferring the varied degree of protonation caused by these acids on the polymer backbone. The direct current (DC) conductivity of protonated and iodine-doped 1,3-polyazulene **5** (0.74 and 1.22 S/cm respectively) was significantly higher than its neutral form (<10^−11^ S/cm). The increased conductivity of 1,3-polyazulene **5** upon protonation can be attributed to the better stabilization of cation radicals, di-, and polycations by the unique dipolar nature of azulene, and in the case of iodine doping, it was attributed to the strengthened spin–spin interaction arising due to a high radical concentration. Recently, it was also established that the water-dispersible poly(1,3-azulene)-polystyrenesulfonate (PSS) polymer can display an overall conductivity up to 0.1 S/cm under ambient conditions and an ion Seebeck coefficient value as high as 4.5 mV K^−1^ [21].

**Scheme 4:**
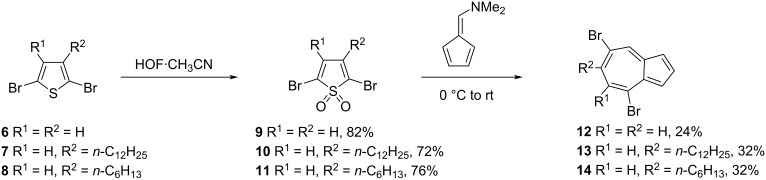
Synthesis of 4,7-dibromo-6-(*n*-alkyl)azulenes **12**–**14**.

In 2012, Hawker and co-workers [22] reported the synthesis of 4,7-polyazulenes **17–20** having backbone connectivity through the seven-membered ring of azulene. The key precursors required in this synthesis, 4,7-dibromo-6-(*n*-alkyl)azulenes **12–14** were synthesized by treating 2,5-dibromo-3-alkylthiophenes **6–8** with HOF·CH_3_CN followed by reaction with dimethylaminofulvene (Scheme 4).

**Scheme 5:**
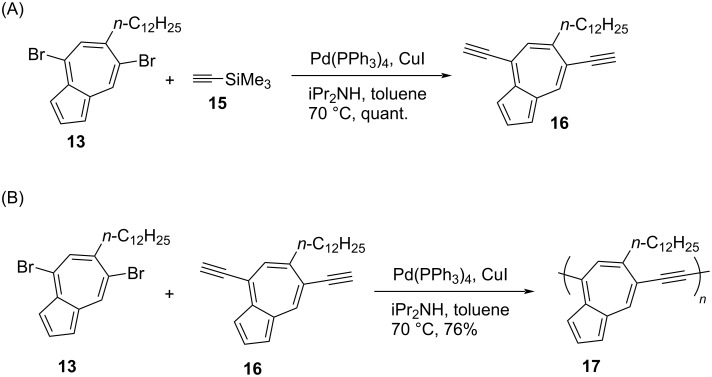
Synthesis of (A) 4,7-diethynyl-6-(*n*-dodecyl)azulene (**16**) and (B) 4,7-polyazulene **17** containing an ethynyl spacer.

**Scheme 6:**
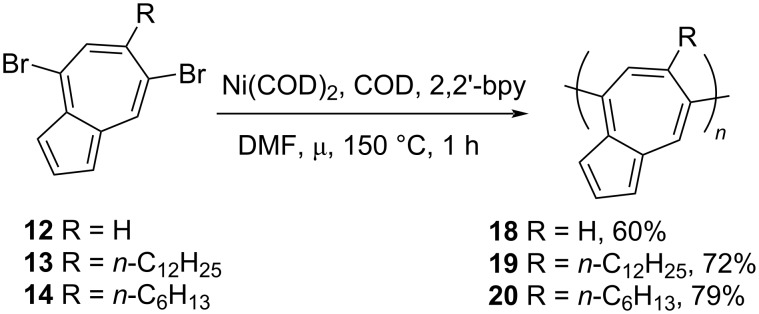
Synthesis of directly connected 4,7-polyazulenes **18–20**.

The synthesis of 4,7-diethynyl-6-(*n*-dodecyl)azulene (**16**) is shown in Scheme 5A. The Sonogashira cross-coupling reaction between 4,7-dibromo-6-(*n*-dodecyl)azulene (**13**) and 4,7-diethynyl-6-(*n*-dodecyl)azulene (**16**) yielded 4,7-polyazulene **17** linked through ethynyl bridges (Scheme 5B). Similarly, the Yamamoto cross-coupling reaction carried out on **12–14** yielded the directly connected 4,7-polyazulenes **18–20** in 60–79% yields (Scheme 6).

The majority of these polymers were soluble in common organic solvents and the GPC data obtained in chloroform showed their *M*_n_ to be in the range of 2.4 to 7.2 KDa. These polymers possessed high thermal stability as well. The absorption maxima displayed by polymer **18** (409 nm) in dichloromethane solution was comparable to the above-discussed 1,3-polyazulene **5** (404 nm), however, the long alkyl substituents present at the 6-position of **19** and **20** were disrupting the effective π-conjugation along the polymer backbone, resulting in the blue shift of absorption bands (λ_max_ around 350 nm). Interestingly, the ethynyl linker in **17** was assisting the effective delocalization despite the presence of the *n*-alkyl group at position 6, as evidenced by its large red-shifted absorption at 456 nm compared to **19**. The protonation of **17** and **18** by TFA resulted in a large red-shifted broad absorption band in the 500–900 nm region due to the formation of azulenium cations in the polymer backbone unlike the 1,3-polyazulene **5**, which had no significant effect on its absorption spectrum upon protonation. The HOMO–LUMO gap for the protonated **17** (1.32 eV) was also reduced compared to its neutral form (1.49 eV). These polymers, unlike 1,3-polyazulene **5**, displayed reversible acid–base chemistry. With the help of absorption spectroscopy and EPR analysis, the authors could establish the fact that effective stabilization of 6 π-electron tropylium cations can be achieved by incorporating the seven-membered rings of azulene in the polymer backbone. The enhanced stability of these 4,7-polyazulenes **17–20** in acids and their stimuli-responsible behavior make them potential materials for photonic device applications.

#### Poly[2,6-aminoazulene]

**Scheme 7:**
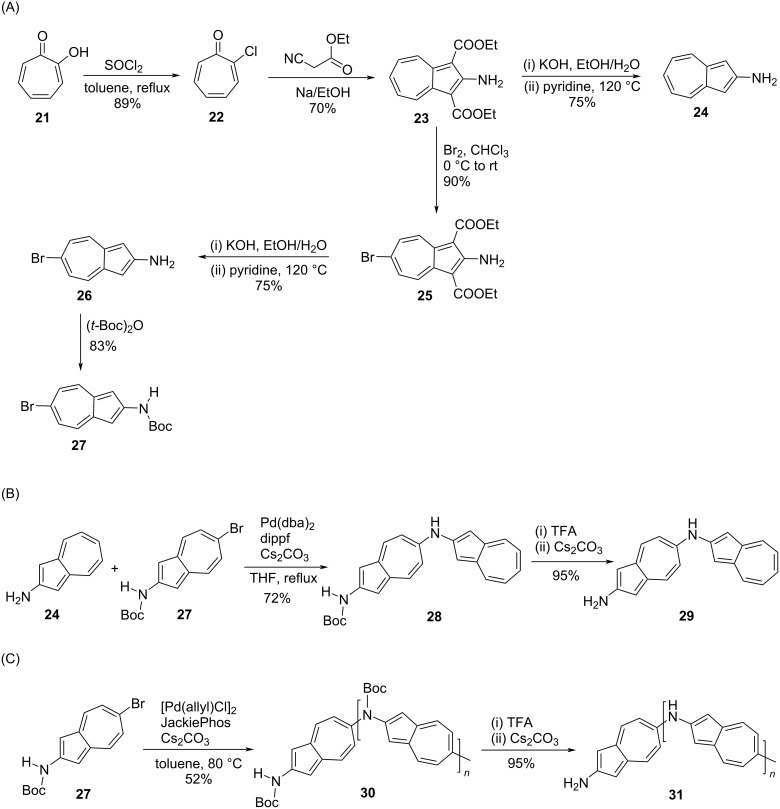
Synthesis of (A) *tert*-butyl *N*-(6-bromoazulen-2-yl)carbamate (**27**), (B) dimeric aminoazulene **29**, and (C) poly[2,6-aminoazulene] **31**.

In order to construct polyazulenes with the head-to-tail alignment of dipoles, azulene should be functionalized at the 2,6-positions, which are diagonally opposite to each other. This will lead to an alternate arrangement of seven and five-membered rings along the polymer backbone and can facilitate effective electron delocalization along the *C*_2_ axis (*C*_2_*_v_* symmetry) passing through 2,6-positions of azulene [23–25]. The first chemical synthesis of such polyazulene called poly[2,6-aminoazulene] **31** was reported by Luh and co-workers [26] in 2017 and achieved by using the Buchwald–Hartwig reaction protocol (Scheme 7). The synthesis of the key building blocks 2-aminoazulene (**24**), 2-amino-6-bromoazulene (**26**), and its corresponding carbamate, *tert*-butyl *N*-(6-bromoazulen-2-yl)carbamate (**27**), used in their synthesis is presented in Scheme 7A. The carbamate **27** was subjected to a Buchwald–Hartwig reaction by using [Pd(allyl)Cl]_2_ and JackiePhos as a ligand to obtain the polymer **30** in 52% yield. The deprotection of the *N*-Boc functionality led to the formation of poly[2,6-aminoazulene] **31** in excellent yields (Scheme 7C).

The *N-*Boc-protected polymer **30** possessed good solubility in organic solvents and its *M*_n_ and PDI were 2900 Da and 1.22, respectively. The aminoazulene dimer **29** was also synthesized (Scheme 7B) in this study as a reference compound. When compared to the dimeric aminoazulene **29** (481 nm) and 2-aminoazulene (**24**, 394 nm), the polyaminoazulene **31** exhibited a far red-shifted broad absorption band spanning 400–800 nm in NMP solution with a maximum at 591 nm. Accordingly, the optical energy gap for the polymer **31** was the lowest (1.65 eV) in comparison to dimer **29** (2.15 eV) and monomer, 2-aminoazulene (**24**, 3.15 eV) emphasizing the advantage associated with inducing a 2,6-connectivity along the polymer backbone.

**Figure 2:**
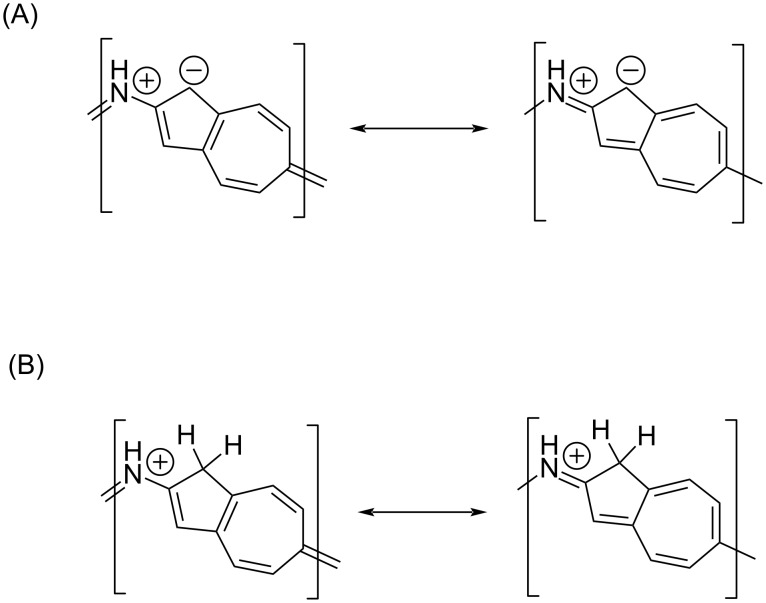
Iminium zwitterionic resonance forms of poly[2(6)-aminoazulene] **31**.

The iminium zwitterion resonance structure (Figure 2) can contribute in extending the conjugation along the backbone of poly[2(6)-aminoazulene] **31** and this property of **31** makes it very different from the well-known polyaniline. The absorption peak positions of the protonated forms of monomer **24**, dimer **29**, and polyazulene **31** were further red-shifted and their respective energy gaps were also reduced compared to their neutral forms. The oxidation potential for the neutral (−0.23 V) and protonated forms of polyaminoazulene **31** (0.70 V) was significantly different unlike the case of protonated emeraldine. The Nafion membrane doped with protonated polyaminoazulene **31** displayed good proton conductivity and reduced methanol permeability, making it suitable for methanol fuel cell applications.

### Azulene-containing copolymers

#### Azulene-thiophene/bithiophene copolymers

**Scheme 8:**
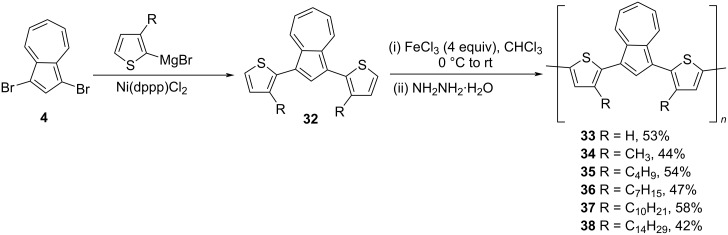
Synthesis of poly{1,3-bis[2-(3-alkylthienyl)]azulene} **33–38**.

Lai and co-workers [27–28], in 2004, reported the synthesis of conjugated polymers containing azulene and bithiophene units, called poly{1,3-bis[2-(3-alkylthienyl)]azulene} **33–38**. The 1,3-bis(3-alkylthienyl)azulene **32**, synthesized by the Grignard reaction of 1,3-dibromoazulene (**4**) with 2-bromo-3-alkylthiophene, was subjected to the oxidative polymerization by using ferric chloride to obtain polymers **33–38** in 42–58% yields as shown in Scheme 8.

These polymers possessed good thermal stability (*T*_d_ > 400 °C) and most of them were soluble in organic solvents such as THF, chloroform, dichloromethane, xylene, and toluene. The number-average molecular weights (*M*_n_) of polymers **33–38** were in the range 16,000 to 41,000 Da (determined by GPC in THF) and the degree of polymerization was 40–60. The conjugation between the azulene-bithiophene units along the polymer backbone resulted in the red-shifted absorption bands compared to their corresponding monomers and, upon protonation, the absorption bands were even extended to the NIR region [29]. The conductivity studies performed on either iodine-doped (*p*-doping or oxidative doping) or protonated polymers (by using TFA) showed higher conductivities in comparison to most of the poly(thiophene-arylene) copolymers [30–31], emphasizing the role of the azulene units in stabilizing the polarons or bipolarons formed during the doping process. In particular, among the iodine-doped polymers, **38** displayed a maximum conductivity of 2.23 S/cm with 45% iodine uptake, and the protonated polymer **36** recorded a conductivity value of 1.10 S/cm. The electrochemical impedance spectroscopy (EIS) studies revealed that these polymers could be used as coatings in corrosion control applications as their conductivity was significantly enhanced (10^3^–10^4^-fold) when kept in contact with the aqueous acid solution.

**Scheme 9:**
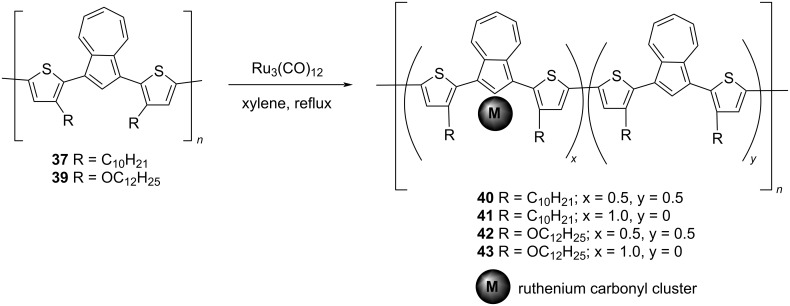
Synthesis of polymer ruthenium complexes **40**–**43**.

Further, the coordination of polymers **37** and **39** to a multinuclear Ru cluster was investigated by the same group [32]. The organometallic complexes **40–43** were synthesized by treating polymers **37** and **39** with Ru_3_(CO)_12_ in refluxing xylene (Scheme 9).

The ruthenium content in these complexes can be varied by changing the ratio of reactants during the reaction. The ^1^H NMR chemical shift values of the azulene unit were used as a tool to determine the extent of ruthenium coordination to the azulene units in the polymer backbone as the coordinated azulene displays upfield-shifted resonance signals when compared to free azulenes. The absorption and electrochemical studies conducted on these complexes **40–43** revealed that their properties could be tuned by varying the ruthenium content in the polymer. A higher ruthenium content was inducing a larger blue-shift of the absorption bands and larger cathodic shift of the oxidation potential in these polymers compared to their metal-free counterparts.

**Scheme 10:**
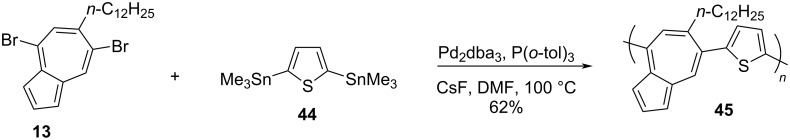
Synthesis of 4,7-polyazulenes **45** containing a thienyl linker.

In 2012, Hawker and co-workers [22], along with **17–20**, also synthesized a polymer **45** which had azulene and thiophene units connected via the 4,7-positions of azulene. The reaction of 4,7-dibromo-6-(*n*-dodecyl)azulene (**13**) with 2,5-bis(trimethylstannyl)thiophene (**44**) under Stille reaction conditions produced the polymer **45** in 62% yield (Scheme 10).

The *M*_n_ and PDI for polymer **45** were 5100 Da and 1.4, respectively and its thermal stability was excellent (*T*_d_ = 404 °C). Polymer **45** exhibited reversible acid–base response, which was evident from its absorption and EPR studies. The optical HOMO–LUMO gap for **45** was smaller in the protonated form (1.50 eV) compared to its neutral form (1.62 eV).

**Scheme 11:**
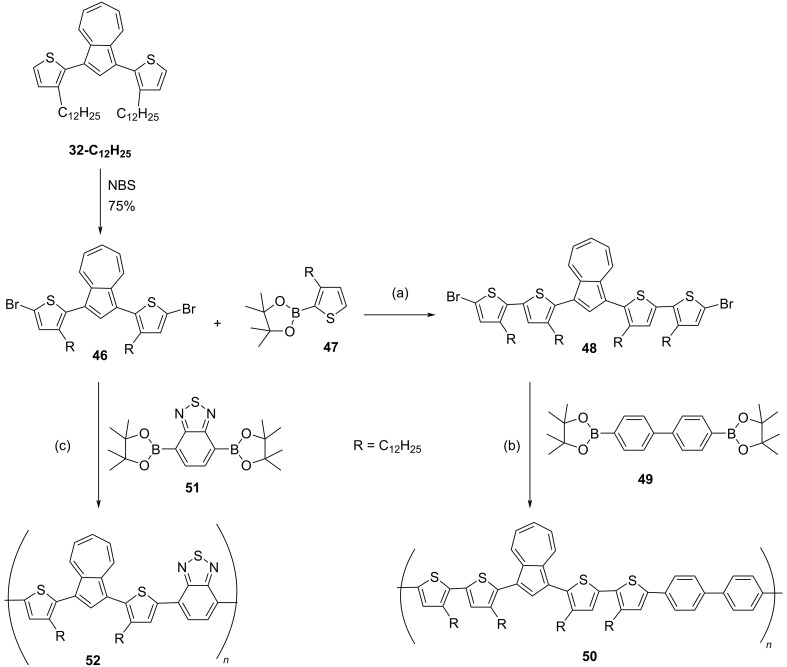
Synthesis of azulene-bithiophene **48** and azulene-benzothiadiazole **52** copolymers. Conditions: (a): (i) Na_2_CO_3_, Aliquat 336, Pd(PPh_3_)_4_, 100 °C, toluene, 90% (ii) NBS, CHCl_3_/HOAc, −10 °C, 83%, (b): Na_2_CO_3_, Aliquat 336, Pd(PPh_3_)_4_, toluene, 110 °C, 9%, (c): Na_2_CO_3_, Aliquat 336, Pd(PPh_3_)_4_, toluene, 110 °C, 12%.

**Scheme 12:**
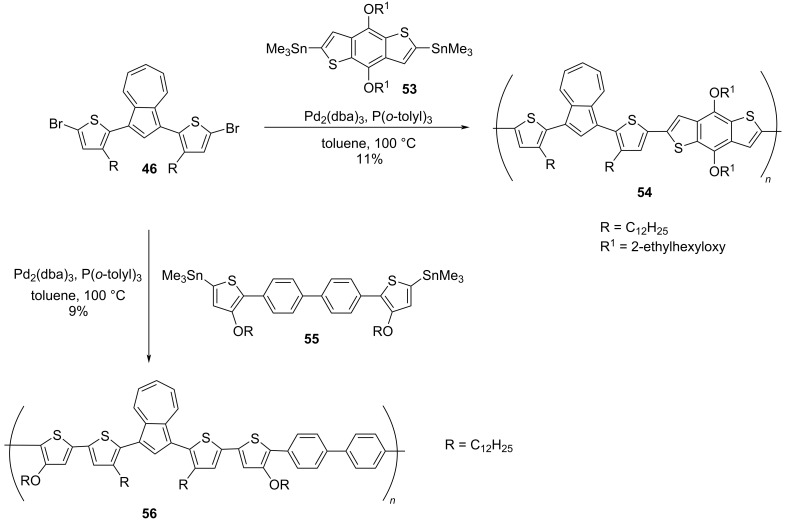
Synthesis of azulene-benzodithiophene copolymer **54** and azulene-bithiophene copolymer **56**.

In 2014, Wang, He, and co-workers [33–34] synthesized the conjugated polymers containing poly(thienyl-azulene) units capable of absorbing in the near IR region (1.0 to 2.5 μm). Their synthetic strategy involved employing 1,3-dibromo-[2-(3-dodecylthien-2-yl)]azulene (**46**) as a key precursor and Suzuki and Stille cross-coupling reactions as polymerization tools. The polymer **50** was synthesized from the 1,3-dibromo-[2-(3-dodecylthien-2-yl)]azulene (**46**) by using two successive Suzuki coupling reactions with thiophene boron ester **47** and biphenyl-4,4’-diboronic acid bis(pinacol) ester **49** (Scheme 11), whereas the Suzuki coupling of **46** with 2,1,3-benzothiadiazole-4,7-bis(boronic acid pinacol ester) **51** yielded the polymer **52** in good yields (Scheme 11). The other two polymers **54** and **56** were synthesized through Stille coupling reactions of **46** with 2,6-bis(trimethylstannyl)-4,8-bis(2-ethylhexyloxy)benzo[1,2-*b*:4,5-*b*’]dithiophene (**53**) and tin agent **55**, respectively, as shown in the Scheme 12.

These polymers **50**, **52**, **54**, and **56** displayed good solubility in most of the commonly used organic solvents and GPC analysis revealed their number-average molecular weight (*M*_n_) to be in the range of 25800 to 48600 Da with polydispersity in the range 1.29–1.9. They also exhibited excellent thermal stability as indicated by their decomposition temperature, which was over 350 °C. The polymers **50**, **54**, and **56** exhibited absorption bands in the 400–450 nm range due to π–π* transition. However, polymer **52**, which contains a benzothiadiazole ring was an exception, as it showed a highly red-shifted band at 550 nm. The important feature of these polymers worth highlighting here is the gradual bleaching of bands due to π–π* transition and display of absorption bands in the near-IR region upon protonation with TFA. The protonated forms of these polymers displayed absorption in the region 1900–2500 nm, i.e., almost covering the entire near-IR region of the spectrum, the largest shift of 2500 nm was displayed by the polymer **56**. The origin of such absorption bands in the near-IR region can be attributed to an intramolecular charge transfer (ICT) process leading to the lowering of the HOMO–LUMO bandgap [34]. The azulene moiety plays a pivotal role here as it is polar resonance stabilized and protonation leads to a drift in its electron density from the seven-membered ring to the five-membered ring generating a stable tropylium cation making it a strong electron acceptor during the intramolecular charge transfer (ICT) process. These protonated polymers were very stable in solution for a long period of time. All these polymers exhibited good chemical and electrochemical stability and easy film-forming properties, important attributes for fabricating near-IR-based optoelectronic devices.

**Scheme 13:**
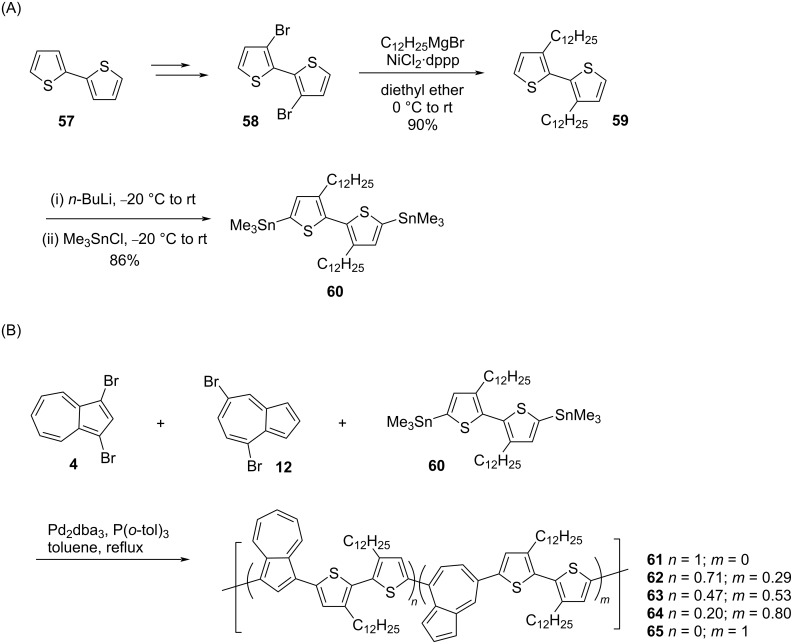
Synthesis of (A) 5,5’-bis(trimethylstannyl)-3,3’-didodecyl-2,2’-bithiophene (**60**) and (B) azulene-bithiophene copolymers **61–65** containing varied ratios of different regioisomers of azulene.

In 2014, Hawker, Robb, and co-workers [35] reported the synthesis of azulene-bithiophene copolymers **61–65** in which varied ratios of different regioisomers of azulene (1,3- and 4,7-connected) were present (Scheme 13). The synthesis of the key building block 5,5’-bis(trimethylstannyl)-3,3’-didodecyl-2,2’-bithiophene (**60**) is shown in Scheme 13A. The reaction of the bis-stannylated bithiophene **60** with varying ratios of dibromoazulenes **4** and **12** under Stille reaction conditions furnished the azulene-bithiophene copolymers **61–65** in decent yields (Scheme 13B).

The final composition of the 1,3- and 4,7-regioisomers of azulene in the polymer chain was determined by ^1^H NMR spectroscopy. All these polymers **61**–**65** were soluble in common organic solvents like THF, dichloromethane, and chloroform and had *M*_n_ in the range 6000 to 21000 Da with PDI of 1.3–1.6. The polymers **61**–**65** displayed stimuli-responsive behavior as evidenced by shifting of their absorption bands from the UV region to the near-IR region upon protonation. This property was dependent on the composition of the azulene units and their connection pattern along the polymer backbone. For example, the protonated polymer **62** with 71% 1,3-disubstituted azulene displayed the highest absorption in the near-IR region. Also, the protonated polymer **61** showed strong fluorescence at 480 nm with a large Stokes shift of 155 nm.

**Scheme 14:**
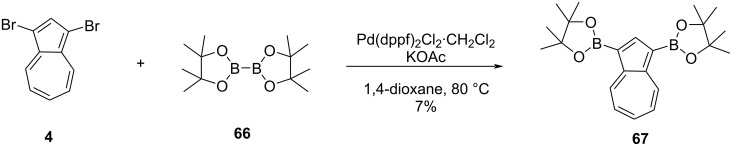
Synthesis of 1,3-bisborylated azulene **67**.

**Scheme 15:**
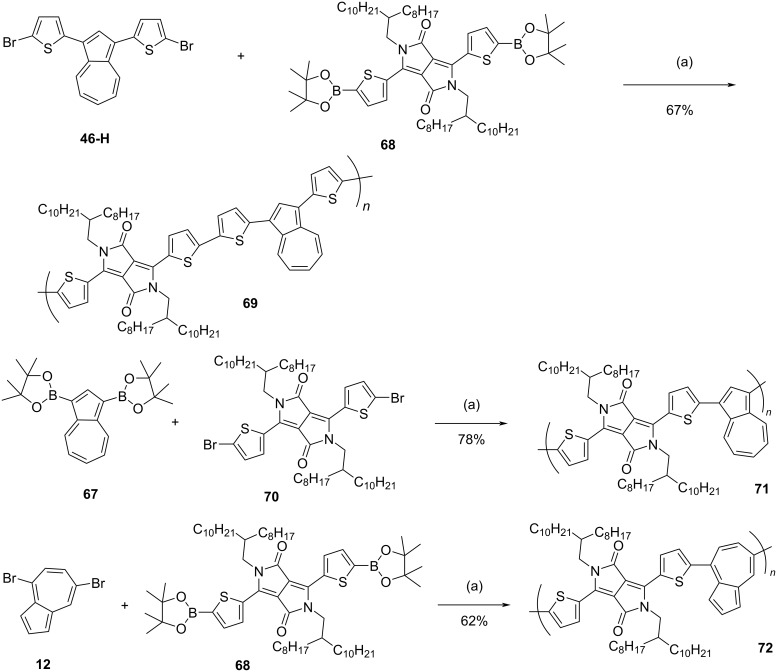
Synthesis of D–A-type azulene-DPP copolymers **69**, **71**, and **72**. Conditions: (a) Pd(PPh_3_)_4_, K_2_CO_3_, Aliquat 336, toluene, 90 °C.

In 2015, Zhang, Liu, and co-workers [36] reported the synthesis of conjugated donor–acceptor (D–A)-type azulene-dithienyldiketopyrrolopyrrole (DPP) polymers **69**, **71**, and **72**. The synthesis of one of the key building blocks, 1,3-bisborylated azulene **67** is shown in the Scheme 14. The azulene-DPP polymers containing azulene units connected via 1,3- and 4,7-positions are synthesized in decent yields by using appropriately functionalized azulene and DPP monomers under Suzuki reaction conditions as delineated in Scheme 15.

All these polymers were soluble in organic solvents such as toluene, chloroform, tetrachloroethane, and the *M*_w_ for **69**, **71**, and **72** were 41700, 38100, and 49400 Da with PDI being 3.4, 2.7, and 3.3, respectively. The polymers were thermally stable with *T*_d_ above 300 °C. The absorption spectra of **69**, **71**, and **72** in chloroform displayed strong absorption at 667, 670, and 627 nm, and in the thin-film form they were further red-shifted. The optical energy gaps for **69**, **71**, and **72** were 1.33, 1.38, and 1.23 eV, respectively. Thin-films of **69** and **71**, in which azulene was incorporated onto the polymer backbone in a 1,3-fashion, exhibited p-type semiconducting behavior, whereas the polymer **72**, in which a 4,7-connectivity pattern of azulene is present, was ambipolar with hole and electron mobilities of 0.062 and 0.021 cm^2^ V^−1^ s^−1^, respectively. The polymer **69** behaved like an electron donor for organic PV cells and the blend of thin-film **69** with PC_71_BM showed a power conversion efficiency (PCE) of 2.04%.

**Scheme 16:**
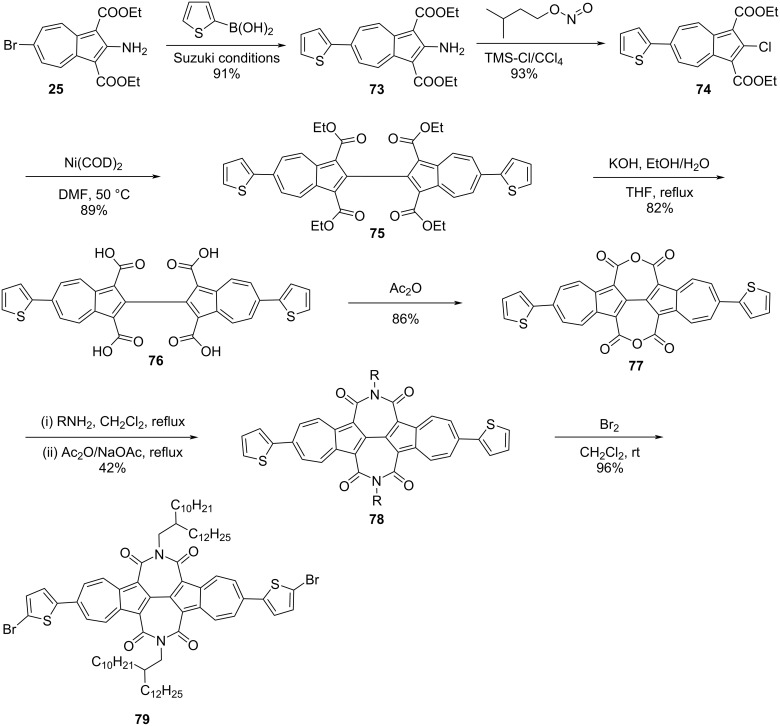
Synthesis of the key precursor TBAzDI **79**.

In 2018, Gao and co-workers [37] reported the synthesis of conjugated polymers containing 2,6-connected azulene units in the polymer backbone. The key monomer, *N*,*N*’-bis(2-decyltetradecyl)-6,6’-di(thiophen-2-yl)-[2,2’-biazulene]-1,1’3,3’-tetracarboxdiimide (TBAzDI) **79** to make these polymers was synthesized as shown in the Scheme 16.

**Scheme 17:**
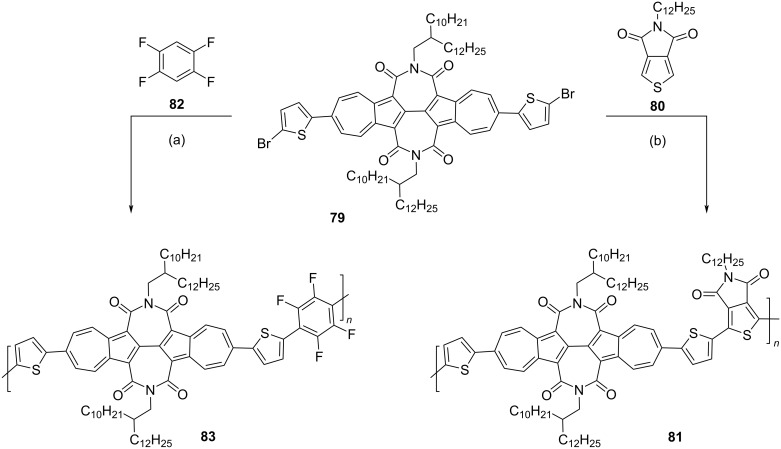
Synthesis of TBAzDI-based polymers **81** and **83**. Conditions: (a) P(*o*-tol)_3_, Pd_2_(dba)_3_, PivOH, Cs_2_CO_3_, THF, 80 °C, 68%; (b) P(*o*-tol)_3_, Hermann's catalyst, Cs_2_CO_3_, THF, 120 °C, 77%.

The monomer **79** was reacted with *N*-dodecylthieno[3,4-*c*]pyrrole-4,6-dione (TPD, **80**) and 1,2,4,5-tetrafluorobenzene (TFB, **82**) via a palladium-catalyzed direct C–H arylation strategy to obtain the polymers **81** and **83**, respectively, in good yields (Scheme 17).

Both polymers **81** and **83** were soluble in solvents such as chloroform and chlorobenzene and their *M*_n_ and PDI were found to be 22100 and 13500 Da, and 3.68 and 3.26, respectively. Their thermal stability was excellent as they had *T*_d_ above 400 °C. The solution-state absorption spectra of the polymers **81** and **83** in *o*-dichlorobenzene displayed a strong absorption band at 661 and 516 nm, respectively. The thin-film transistors made out of these polymers displayed electron mobility of 0.42 cm^2^ V^−1^ s^−1^ for **81** and 0.24 cm^2^ V^−1^ s^−1^ for **83**. The polymer **81** with an electron mobility of 0.42 cm^2^ V^−1^ s^−1^ represents one of the best unipolar n-type polymers for OFET applications. These polymers were also tested as electron acceptors for all-polymer solar cell (PSC) devices, and **81** in particular, showed a power conversion efficiency (PCE) value of 1.82%. These results reiterated the importance of the 2,6-connectivity pattern of azulene units along the polymer backbone.

**Scheme 18:**
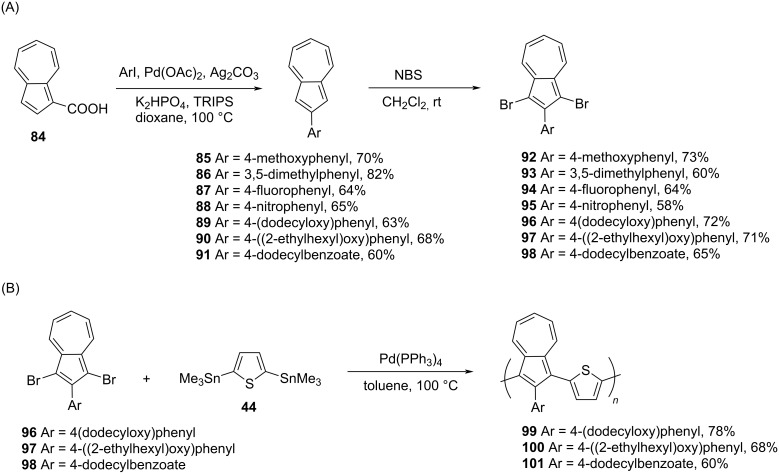
Synthesis of (A) 1,3-dibromo-2-arylazulene **92–98** and (B) 2-arylazulene-thiophene copolymers **99–101**.

Very recently, Xu and Png [38] reported azulene-thiophene copolymers, poly(2-arylazulene-*alt*-thiophene) **99**–**101**, in which the 2-position of the azulene was substituted by an aryl group. To achieve the synthesis of these polymers, 1,3-dibromo-2-arylazulenes **92**–**98**, were first synthesized from azulene-1-carboxylic acid (**84**) in two steps as shown in Scheme 18A [39]. The treatment of dibromo monomers **96–98** with bis(trimethylstannyl)thiophene (**44**) under Stille reaction conditions yielded poly(2-arylazulene-*alt*-thiophene) **99–101** in good yields (Scheme 18B).

All three polymers possessed good thermal stability and their *M*_n_ was in the range of 6900 to 12000 Da. Their absorption maxima were around 350 nm in chloroform solution and were red-shifted (up to 1000 nm) on protonation. The electrochemical HOMO–LUMO gap for polymers **99**–**101** was in the range 1.2–1.35 eV. The polymers **99** and **100** having an electron-donating aryl group at the 2-position were most sensitive to acid (TFA) and changed their colors drastically. This enhanced acid sensitivity could be attributed to the resonance stabilization of the positive charge on the five-membered ring of the azulene by the electron-donating groups at the 2-position. These polymers were quite robust as they underwent several cycles of electro-cycling before degradation.

#### Azulene-fluorene copolymers

**Scheme 19:**
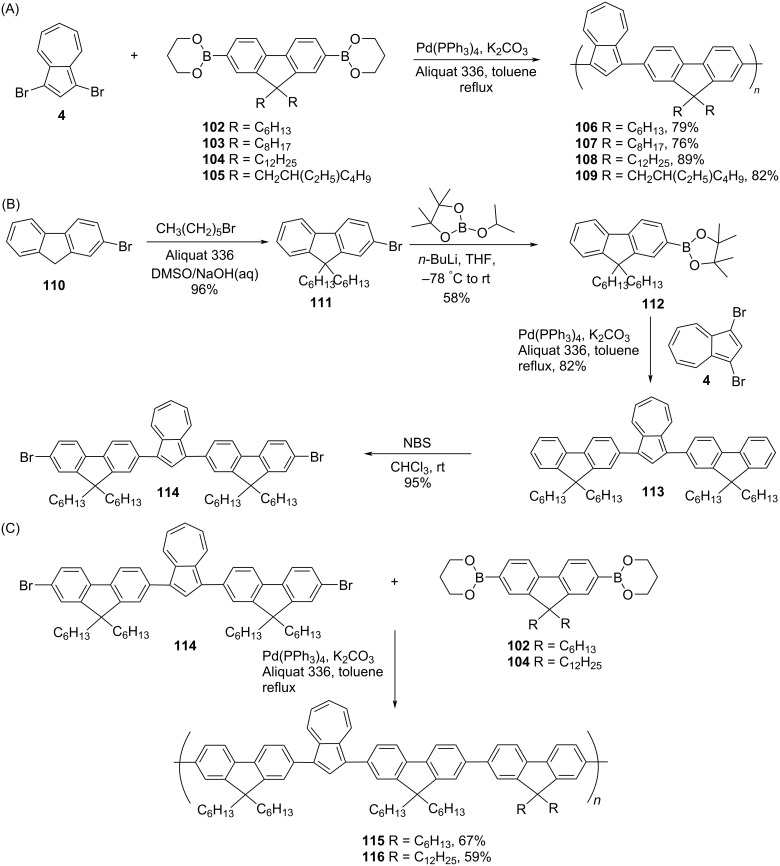
Synthesis of (A) poly[2,7-(9,9-dialkylfluorenyl)-*alt*-(1’,3’-azulenyl)] **106–109**, (B) 1,3-bis(7-bromo-9,9-dihexylfluoren-2-yl)azulene (**114**) and (C) poly{[1,3-bis(9,9’-dihexylfluoren-2’-yl)azulenyl]-*alt*-[2”,7”-(9”,9”-dialkylfluorenyl]} **115** and **116**.

In 2009, Xu and co-workers [40] synthesized various examples of azulene-fluorene conjugated polymers by using Suzuki cross-coupling reactions. The first set of polymers, poly[2,7-(9,9-dialkylfluorenyl)-*alt*-(1’,3’-azulenyl)] **106**–**109** was prepared by the reaction of 1,3-dibromoazulene (**4**) with 9,9-dialkylfluorene-2,7-bis(trimethyleneborate) **102**–**105** under Suzuki reaction conditions (Scheme 19A). To make the other two polymers, poly{[1,3-bis(9,9’-dihexylfluoren-2’-yl)azulenyl]-*alt*-[2”,7”-(9”,9”-dialkylfluorenyl]}, **115** and **116**, a key azulene-containing building block, 1,3-bis(7-bromo-9,9-dihexylfluoren-2-yl)azulene (**114**) was required, and its synthesis was achieved from 2-bromofluorene (**110**) by following the protocol presented in Scheme 19B. The reaction of **114** with fluorene borates **102** and **104** under Suzuki conditions yielded polymers **115** and **116**, respectively (Scheme 19C).

The solubility of all polymers was good in organic solvents like THF, dichloromethane, chloroform, and toluene, and the thermal data reflected on their high thermal stability (*T*_d_ 408–434 °C). The number average molecular weight *M*_n_ (GPC in THF) and polydispersity of these polymers were in the range of 6800–12500 Da and 1.29–2.21, respectively. The UV–vis absorption features of polymers **115** and **116** were altered upon protonation due to the formation of azulenium cations in the polymer backbone and they displayed a significant color change on protonation. None of these polymers were fluorescent in the neutral form, however, **115** and **116** displayed fluorescence emission at 385 nm in the protonated state. The electrochemical bandgap of these polymers was in the impressive range of 1.57–1.62 eV.

**Scheme 20:**
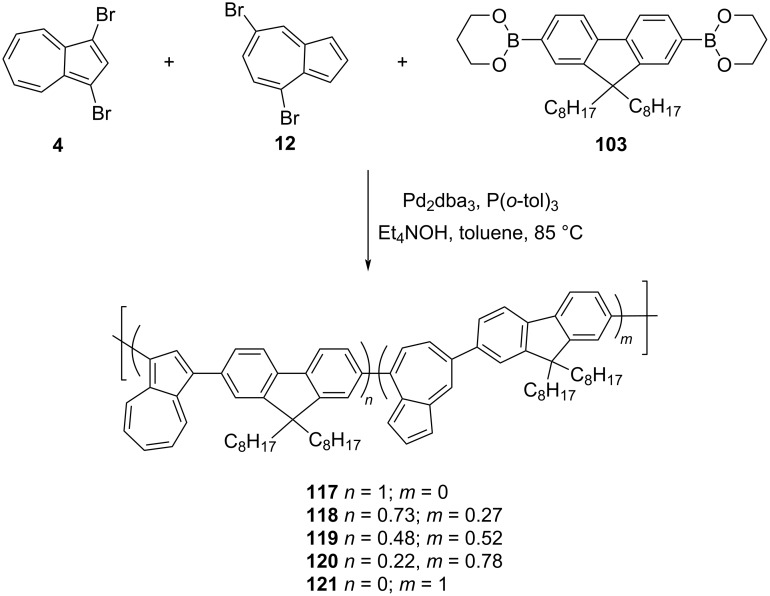
Synthesis of azulene-fluorene copolymers **117–121** containing varying ratios of 1,3- and 4,7-connected azulene units.

In 2014, Hawker, Robb, and co-workers [35] also reported the synthesis of azulene-fluorene copolymers **117**–**121**, containing varying ratios of 1,3-and 4,7-connected azulene units along the polymer chain (Scheme 20). The key building blocks used in this synthesis were 1,3-dibromoazulene (**4**), 4,7-dibromoazulene (**12**), and 9,9-dioctylfluorene-2,7-diboronate **103**. The synthesis of the polymers **117**–**121** were carried out by reacting 9,9-dioctylfluorene-2,7-diboronate **103** with varying ratios of 1,3-dibromoazulene (**4**) and 4,7-dibromoazulene (**12**) under Suzuki reaction conditions (Scheme 20).

As stated above in the case of azulene-thiophene polymers **61**–**65**, the final composition of 1,3- and 4,7-regioisomers of azulene in the polymer chain was determined by ^1^H NMR spectroscopy. All these polymers were soluble in common organic solvents like THF, dichloromethane, and chloroform, and had *M*_n_ in the range 9800 to 34300 Da with PDI of 1.6–2.2. The absorption studies conducted on these polymers revealed their stimuli-responsive nature in protonated states and the influence of the substitution pattern of the azulene units on this behavior. The polymer containing entirely 1,3-substituted azulene, **117**, exhibited a strong fluorescence at 420 nm in the protonated state and the attenuation of the fluorescence signals took place with the increasing population of 4,7-disubstitued azulene units in the polymer chain. The thin film made up of polymer **121**, containing entirely 4,7-disubstituted azulene, showed a reversible and rapid color switching in an acidic environment. This paper presented an interesting strategy of tuning the property of a polymer by incorporating a varying degree of regioisomers of same building block.

**Scheme 21:**
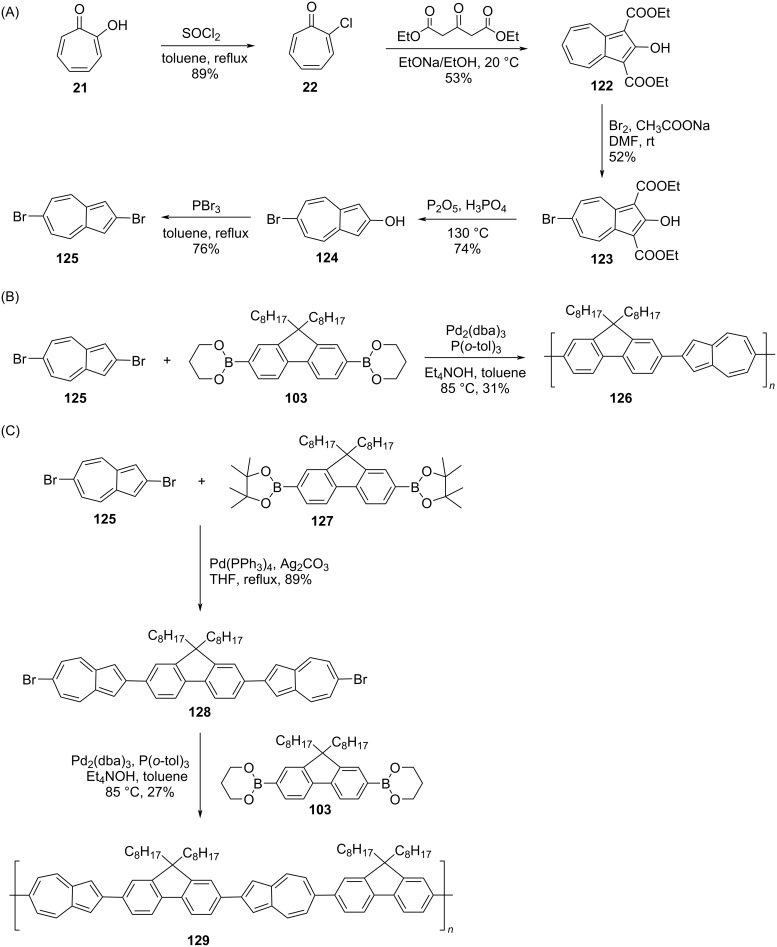
Synthesis of (A) 2,6-dibromoazulene (**125**), (B) azulene-fluorene copolymer **126**, and (C) azulene-fluorene copolymer **129**.

Further, in 2019, Gao and co-workers [41] disclosed the synthesis of azulene-fluorene conjugated polymers connected via 2,6-positions of the azulene ring (Scheme 21). The key starting material to make these polymers was 2,6-dibromoazulene (**125**), which was synthesized from tropolone (**21**) in five steps (Scheme 21A). The Suzuki–Miyaura coupling reaction of 2,6-dibromoazulene (**125**) with 2,2’-(9,9-dioctyl-9*H*-flourene-2,7-diyl)di(1,3,2-dioxaborolane) (**103**) furnished the polymer **126** in 31% yield (Scheme 21B). The other polymer **129** was obtained from 2,6-dibromoazulene (**125**) in two steps as presented in Scheme 21C.

The two polymers **126** and **129** possessed good solubility in organic solvents and high thermal stability (*T*_d_: 418 and 432 °C for **126** and **129**, respectively in a nitrogen atmosphere). The *M*_n_ and PDI for **126** was 40400 Da and 2.08 and for **129** 58300 Da and 1.73, respectively. The response to protonation (TFA) of these polymers was noteworthy as they exhibited rapid and reversible color changes both in solution and thin-film state. The direct current (DC) conductivity values recorded for the thin films of the protonated polymers **126** and **129** were 2.94 and 0.32 S/cm, respectively, far larger than the value noticed for its protonated 1,3-connected counterpart (10^−3^ S/cm). This is presumably due to the ease of protonation of the 5-membered ring of azulene rings in the polymer as they have vacant 1,3-positions available for protonation.

**Scheme 22:**
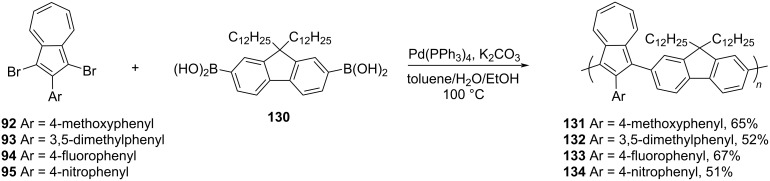
Synthesis of 2-arylazulene-fluorene copolymers **131–134**.

Xu and Png [38], along with the above-mentioned poly(2-arylazulene-*alt*-thiophene) **99**–**101**, also reported the synthesis of the next generation of azulene-fluorene conjugated polymers, **131**–**134** in which the 2-position of azulene was substituted by different aryl groups (Scheme 22). The 1,3-dibromo-2-arylazulenes **92–95** upon reaction with fluorene diboronic acid **130** under Suzuki reaction conditions yielded polymers **131**–**134** in 51–65% yields (Scheme 22).

These polymers **131**–**134** were thermally very stable and exhibited *M*_n_ and PDI in the range 10300–15100 Da and 1.4–2.3, respectively. Like their predecessors **106**–**109**, their electrochemical bandgap was in the range 1.25–1.60 eV. However, the chemical nature of the aryl substituents situated at the 2-position dictated their ease of protonation with TFA. It was observed that electron-donating substituents present at the 2-position facilitated the protonation whereas electron-withdrawing substituents discouraged the protonation.

**Scheme 23:**
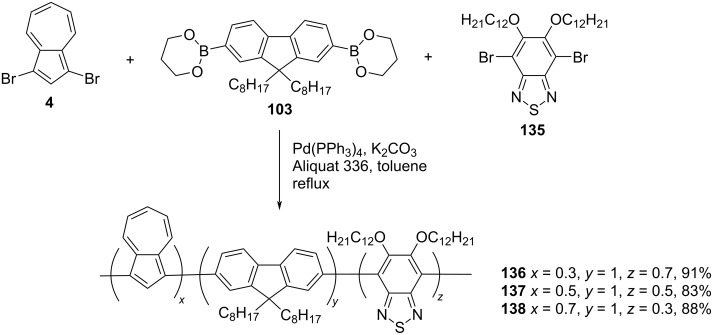
Synthesis of azulene-fluorene-benzothiadiazole terpolymers **136–138**.

In 2013, Xu and co-workers [42] reported the synthesis of azulene-fluorene-benzothiadiazole terpolymers **136–138**, containing varying degrees of azulene and benzothiadiazole units in good yields by adopting the Suzuki coupling protocol (Scheme 23).

The polymers **136–138** were of relatively high molecular weight (*M*_n_ in the range 17000–30900 Da) with PDI 1.45–2.03 and had a good thermal stability (*T*_d_ > 340 °C in N_2_). The solution-state absorption profiles (in THF solution) of these polymers displayed two bands in the region 328–349 and 392–407 nm and these peak positions were red-shifted in comparison to azulene-fluorene polymers because of the electron transfer from the azulene to benzothiadiazole units. The optical (2.19–2.38 eV) and electrochemical (2.25–2.40 eV) band gaps for the polymers **136**–**138** were in good agreement with each other and the gap decreased with an increase in the percentage of azulene in the polymer backbone. The thin films of these polymers exhibited electrochromism, where the color changed from yellowish green (neutral and reduced state) to greyish brown (oxidized state) with electrochromic contrasts of 17 and 13% for polymers **137** and **138**, respectively, in the NIR region. In addition, the azulene-2,1,3-benzothiadiazole containing D–A-type copolymers have also been used in photovoltaic device applications [43].

#### Azulene-carbazole copolymers

**Scheme 24:**
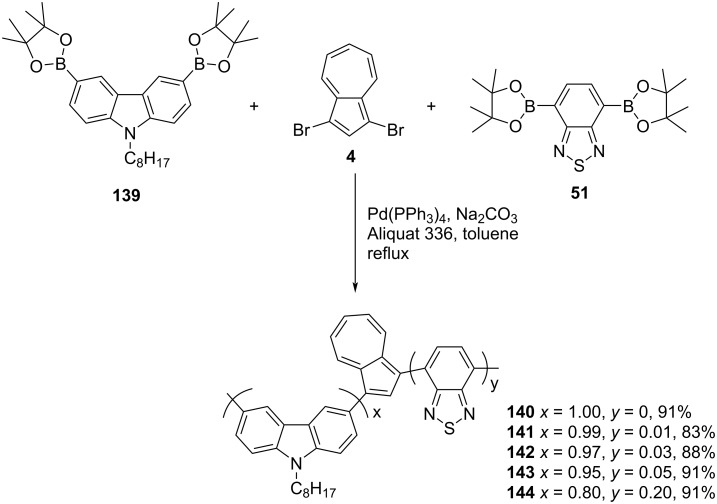
Synthesis of azulene-carbazole-benzothiadiazole-conjugated polymers **140–144**.

The same group [44] also synthesized the azulene-carbazole-conjugated polymer **140** and a set of terpolymers **141**–**144** containing azulene-carbazole-benzothiadiazole, with varying composition of *N*-alkyl carbazole and benzothiadiazole units by a Suzuki protocol (Scheme 24).

These polymers exhibited *M*_n_ in the range 4200–7200 Da with PDI 1.14–1.38. The oxidation potential of the benzothiadiazole-containing polymers **141–144** was low compared to all-azulene-carbazole polymer **140** due to the electron transfer from azulene to benzothiadiazole and, due to this, they exhibited better electrochromism. An electrochromic device (ECD) constructed with polymer **143** exhibited black to transmissive electrochromism with high contrast.

#### Azulene-methacrylate copolymers

**Scheme 25:**
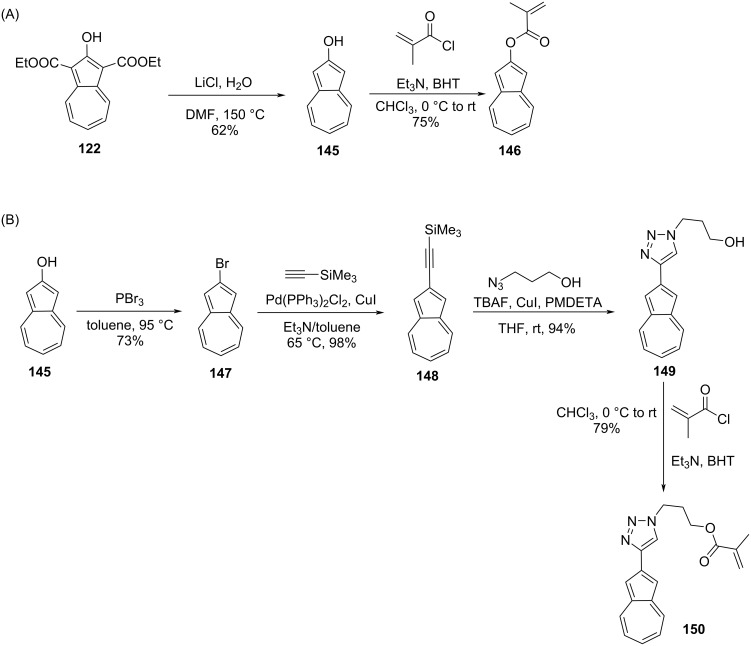
Synthesis of (A) azulene-2-yl methacrylate (**146**) and (B) the triazole-containing azulene methacrylate **150**.

Emrick and co-workers [45] reported the synthesis of azulene-substituted methacrylate polymers derived from a free radical polymerization strategy, where azulenes were used as pendants. The key starting points to make these polymers were azulene-2-yl methacrylate (**146**) and triazole-containing azulene methacrylate **150**, whose synthesis is outlined in Scheme 25. The azulene-2-yl methacrylate (**146**) was synthesized in 75% yield from 2-hydroxyazulene (**145**) by treating it with methacryloyl chloride in chloroform (Scheme 25A). The 2-hydroxyazulene (**145**) was converted to 2-bromoazulene (**147**) and subsequently to the TMS acetylene derivative **148** suitable for the ‘click’ reaction [46] to obtain the triazole **149**, which was eventually transformed into the triazole-containing azulene methacrylate monomer **150** (Scheme 25B).

**Scheme 26:**
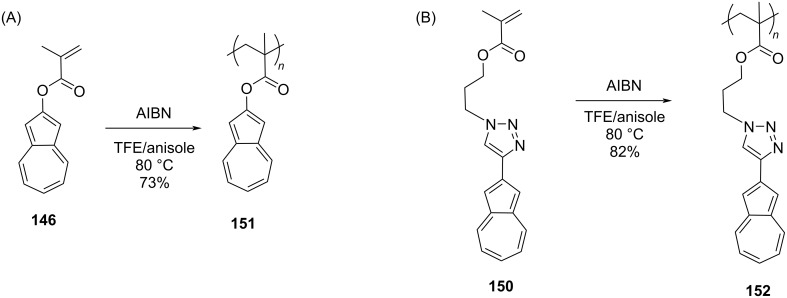
Synthesis of (A) azulene methacrylate polymer **151** and (B) triazole-containing azulene methacrylate polymer **152**.

**Scheme 27:**
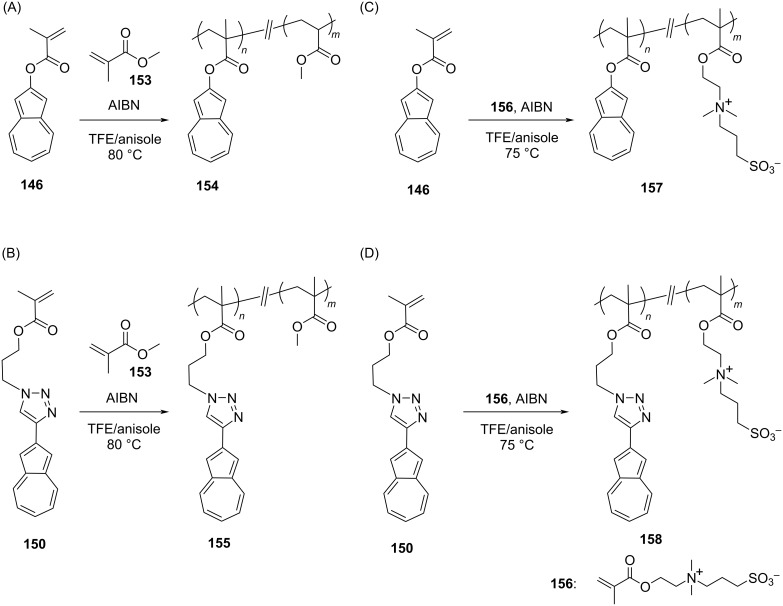
Synthesis of azulene methyl methacrylate polymers **154**, **155** (A and B) and azulene-sulfobetaine methacrylate polymers **157**, **158** (C and D).

The monomers **146** and **150** were then subjected to free radical polymerization by using azobis(isobutyronitrile) (AIBN) to obtain the polymers **151** and **152** in 73 and 82% yields, respectively (Scheme 26A and B). The *M*_n_ and PDI for these polymers **151** and **152** were 13500 Da, 2.5 and 13600 Da, 2.2, respectively, and their solubility was good in organic solvents. In order to obtain polymers containing a varying degree of pendant azulene units, such as **154**, **155**, **157**, and **158**, the authors performed the free radical copolymerization of the monomers **146** and **150** with methyl methacrylate (**153**) (Scheme 27A and B) and sulfobetaine methacrylate (**156**) (Scheme 27C and D) in the presence of AIBN.

The *M*_n_ for the **154** and **155** series was in the range of 13900–22000 Da with a PDI range of 1.7–2.6. Likewise, for the **157** and **158** series the *M*_n_ range was 18900–38900 Da with PDI 1.5–1.7. The composition of azulene in the polymers **154** and **155** was 20–40 mol % azulene, whereas the polymers **157** and **158** were containing 25–75 mol % azulene. The photophysical studies carried out on the neutral/protonated polymer series **155** and **158** were supportive of the fact that the optoelectronic property, electronic interactions, and exciton migration can be tuned by varying the density of pendant azulene units along the polymer backbone. The polymers with increasing density of pendant azulene in the backbone displayed improved device performance in comparison to the control devices featuring only poly(sulfobetainemethacrylate) interlayers. The polymer **158** containing the highest azulene density (75 mol % azulene) was found to be an effective cathode modification layer in a bulk-heterojunction solar cell with a power conversion efficiency of 7.9%.

## Conclusion

This review has described the chemical syntheses and key features of azulene-containing polymers reported in the last three decades. Azulene-containing homopolymers (polyazulenes) and copolymers incorporating thiophene, fluorene, benzothiadiazole, and carbazole units along the polymer backbone can be synthesized by utilizing cross-coupling strategies such as Suzuki, Sonogashira, Stille, Yamamoto, and Buchwald–Hartwig reactions. Azulene can be incorporated onto the polymer backbone by involving its five-membered rings via a 1,3-fashion, the seven-membered rings via a 4,7-fashion and, both the rings can be incorporated via 2,6-fashion, and these patterns can influence the properties of the resulting polymer. By and large, the reported azulene-containing homo- and copolymers have proven to be efficient functional materials for organic field-effect transistor (OFET), all-polymer solar cell (PSC) applications, and they can also exhibit NIR absorption and electrochromism, and electrical conductivity. However, the reports on polymers containing azulene with a 2,6-connectivity pattern are scarce in the literature. As this mode of connectivity pattern has the advantage of involving both the dipoles of azulene onto the polymer backbone, warrants further investigation. The reported 2,6-polyaminoazulene **31** behaved distinctively to polyanilines and can be a useful proton-conducting membrane material in methanol fuel cells. Also, as azulenes are known to contravene Kasha’s rule, it is intriguing to explore, how their presence in the polymers can favor the energy utilization from higher excited states. This may lead to a new dimension in photoluminescent materials research. It can be concluded that the research field of azulene-containing functional polymers is still in its infancy and there is a lot of scope for chemists and material scientists to design and chemically synthesize exotic azulene-containing polymers having improved characteristics. The author hopes that this review might stimulate such research efforts in the future.
